# A Strong Approach to a Weak Gastric Wall in Bariatric Surgery: Concurrent Diverticulectomy and Sleeve Gastrectomy

**DOI:** 10.7759/cureus.6545

**Published:** 2020-01-02

**Authors:** Laura E Wylie, Yadira Villalvazo, Candice Jensen

**Affiliations:** 1 Surgery, University of Arizona College of Medicine, Tucson, USA; 2 Surgery, Banner University Medical Center, Tucson, USA; 3 Surgery, Yuma Regional Medical Center, Yuma, USA

**Keywords:** sleeve gastrectomy, bariatric surgery, gastric diverticula, diverticulectomy

## Abstract

A gastric diverticulum is a rare finding in which the wall of the stomach forms an abnormal sac-like projection. Gastric diverticula can be problematic causing symptoms including but not limited to chronic gastroesophageal reflux, abdominal pain, and bloating. When a gastric diverticulum becomes symptomatic, removal is indicated. In specific cases, laparoscopic gastric diverticula resection can be completed with concurrent bariatric surgery. We report the perioperative approach used in a 34-year-old obese woman with a confirmed symptomatic gastric diverticulum undergoing a gastric diverticulum resection with a concurrent laparoscopic sleeve gastrectomy.

## Introduction

A gastric diverticulum is an outpouching, sac-like protuberance of the stomach. Gastric diverticula are uncommon with a prevalence of 0.04% diagnosed in radiographs with contrast and between 0.01% and 0.11% diagnosed in upper gastrointestinal endoscopy [1-3]. Although most gastric diverticula are asymptomatic and diagnosed by an incidental finding on imaging studies, symptoms such as upper gastrointestinal bleeding, abdominal pain, nausea, bloating, reflux, and dyspepsia may be present [3,4]. The pathophysiology of gastric diverticula can be divided into two hypotheses: congenital and acquired diverticula. It has been proposed that congenital diverticula could form if the gastric fundus herniates through the dorsal mesentery prior to the fusion of the stomach to the posterior body wall during embryogenesis. Acquired diverticula, or pseudodiverticula, usually present secondary to a chronic inflammatory gastrointestinal pathology such as peptic ulcer disease, or a malignancy, which has ultimately caused the gastric wall to weaken, allowing contents to herniate [3]. 

A laparoscopic sleeve gastrectomy (LSG) is a bariatric surgical procedure in which the fundus and greater curvature of the stomach are resected, removing 70%-80% of the stomach’s original volume. Resection of the stomach promotes weight loss by both mechanical and endocrine mechanisms. A smaller gastric volume limits an individual’s ability to consume, promoting weight loss by encouraging a lower caloric intake. Hormones such as ghrelin and glucagon-like peptide are also affected by an LSG. The fundus of the stomach is responsible for producing ghrelin, which is a hormone that increases hunger. Therefore, when the fundus is resected, the patient produces less ghrelin, which increases feelings of satiety. Glucagon-like peptide is increased post-LSG, improving insulin sensitivity and glucose tolerance, as well as increasing feelings of satiety [5]. The goal of an LSG is to promote weight loss as well as improve or eliminate weight-related comorbidities such as type 2 diabetes mellitus, hypertension, hypercholesterolemia, sleep apnea, and joint degeneration.

Few cases have been reported of laparoscopic gastric diverticula resection with a concurrent LSG. We report a method of treatment of the patient’s symptomatic gastric diverticulum, morbid obesity, and weight-related comorbities with one surgical procedure.

## Case presentation

The patient is a 34-year-old morbidly obese female with a 2.4-cm symptomatic gastric diverticulum confirmed by both esophagogastroduodenoscopy (EGD) and upper gastrointestinal series (UGI). The patient reports chronic gastroesophageal reflux disease (GERD), which is resistant to treatment with proton pump inhibitors. She weighs 260 pounds and is 5 foot 3 inches tall, with a body mass index (BMI) of 46. The patient has obesity-related comorbidities including hypertension, hypercholesterolemia, and fatty liver disease. She has a history of previous abdominal surgeries including a laparoscopic cholecystectomy and a laparoscopy for gynecologic evaluation. She has a family history positive for hypertension. She has no history of smoking, alcohol, or recreational drug use.

Preoperative workup included several studies such as a UGI, EGD, and an abdominal CT with contrast. She had Helicobacter pylori testing, routine preoperative blood work including a complete blood count (CBC) and complete metabolic panel, as well as cardiac, dietary, psychiatric, and pulmonary evaluation and clearance. 

**Figure 1:**
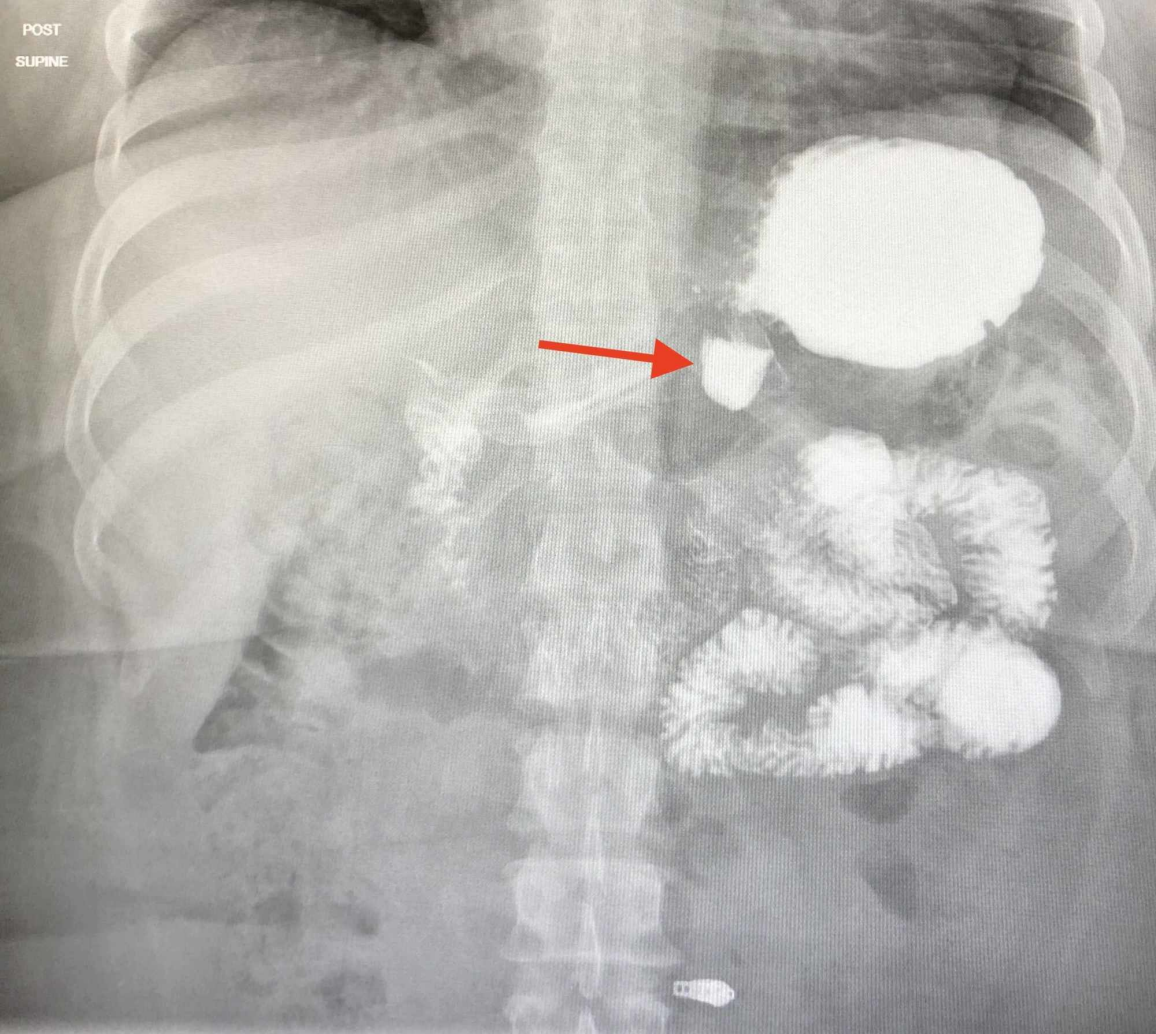
Gastric diverticulum clearly seen in the preoperative UGI. UGI: Upper gastrointestinal series

**Figure 2:**
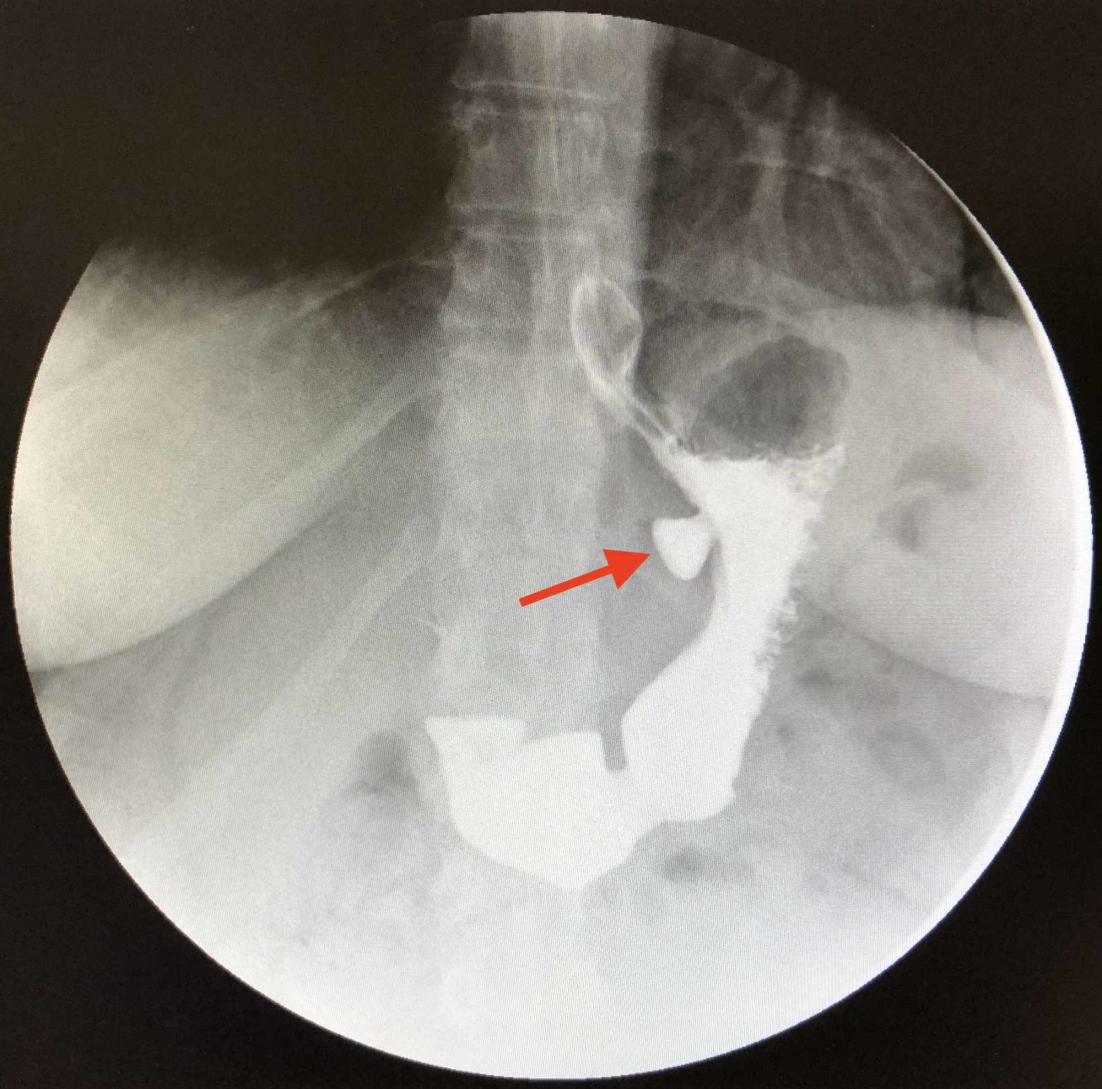
Gastric diverticulum seen as contrast empties into the duodenum during the preoperative UGI. UGI: Upper gastrointestinal series

**Figure 3:**
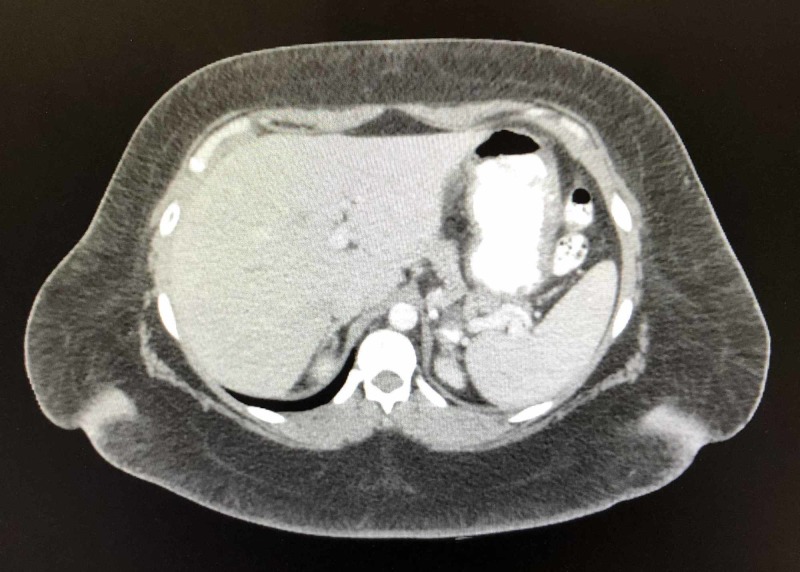
Axial preoperative CT scan was unremarkable.

**Figure 4:**
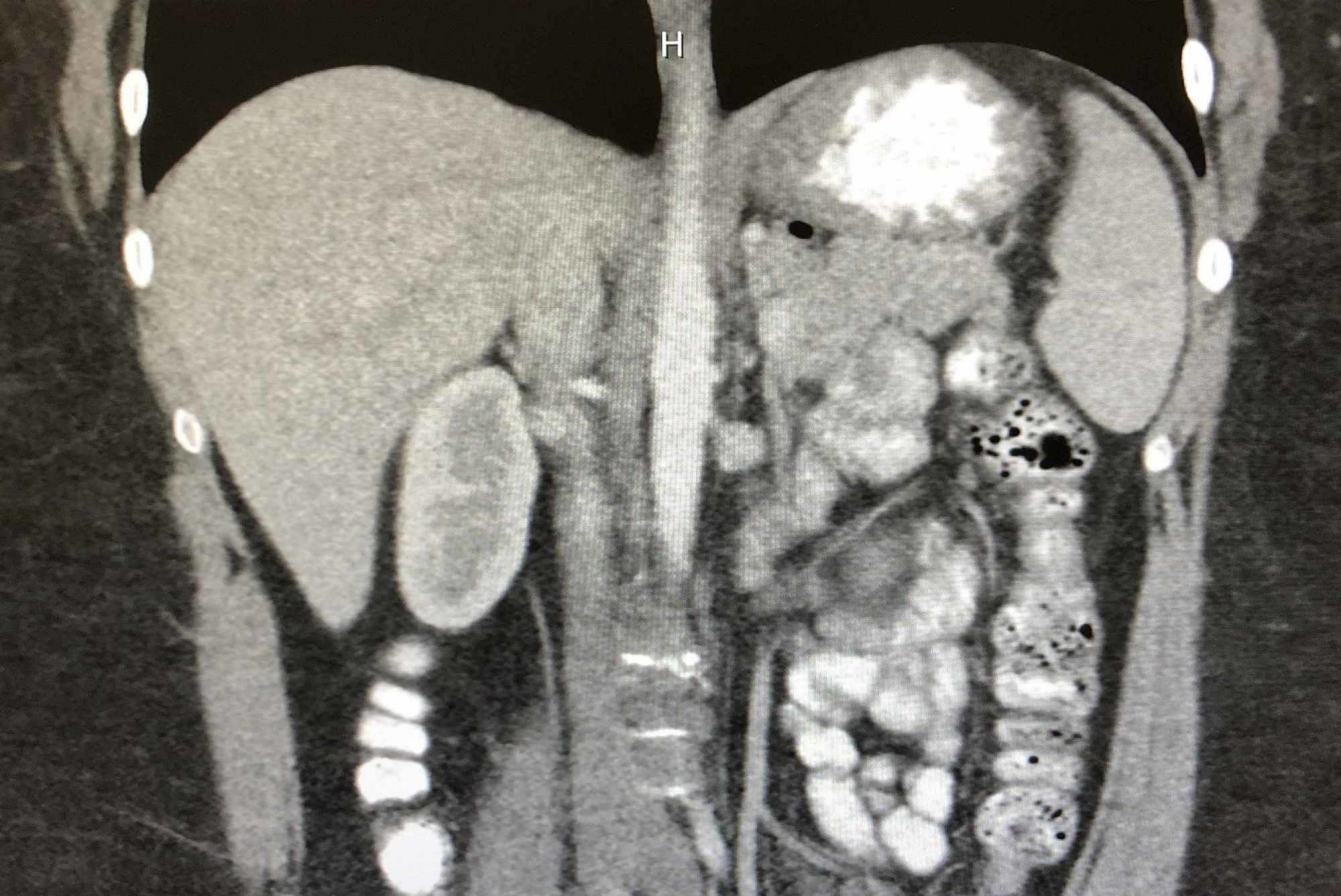
Coronal preoperative CT scan was unremarkable.

The preoperative UGI revealed a small hiatal hernia and a 2.4-cm gastric diverticulum as seen in Figures 1 and 2. EGD confirmed the gastric diverticulum as well as findings of mild antral erythema with mild chronic gastritis (photos not available). The patient’s CT scan was unremarkable as evidenced in Figures 3 and 4. Testing was negative for Helicobacter pylori. Routine blood work results were unremarkable.

The patient lost 20 pounds following a very low calorie liquid diet two weeks prior to surgery. On the day of surgery, the patient received antibiotic prophylaxis with 2 g cefazolin IV 60 minutes prior to surgery. Deep vein thrombosis (DVT) prophylaxis involved administering with 5,000 units of heparin prior to anesthesia, as well placing sequential compression device (SCD) boots bilaterally.

General endotracheal anesthesia was induced with the patient supine. The patient was then prepped and draped, and concurrent diverticulectomy and sleeve gastrectomy were performed in the following way: The abdomen was accessed by placing a Veress needle in the left upper quadrant. Insufflation was created to 15 mmHg. The Veress needle was removed and replaced with a 5 mm trocar and 30 degree laparoscope was placed. Under direct visualization, additional trocars were placed (right and left 5 mm lateral trocars, right and left 15 and 12 mm supraumbilical trocars). The surgeon was positioned on the patient’s right with the assistant on the patient’s left side.

The operating table was placed in reverse Trendelenburg position. The left lobe of the liver was retracted cephalically using a Nathanson. An orogastric calibration tube was then placed, the stomach was decompressed, and the orogastric calibration tube was removed and discarded. An Ethicon Harmonic scalpel was used to incise the peritoneum over the cardia. The plane between the cardia and the left crus of the diaphragm was bluntly dissected to expose the left diaphragmatic crus. The area was examined, and it was determined that no hiatal hernia was present. The Harmonic scalpel was then used to open the bare area over the liver. The lesser curvature of the stomach was examined; no abnormality was seen.

**Figure 5:**
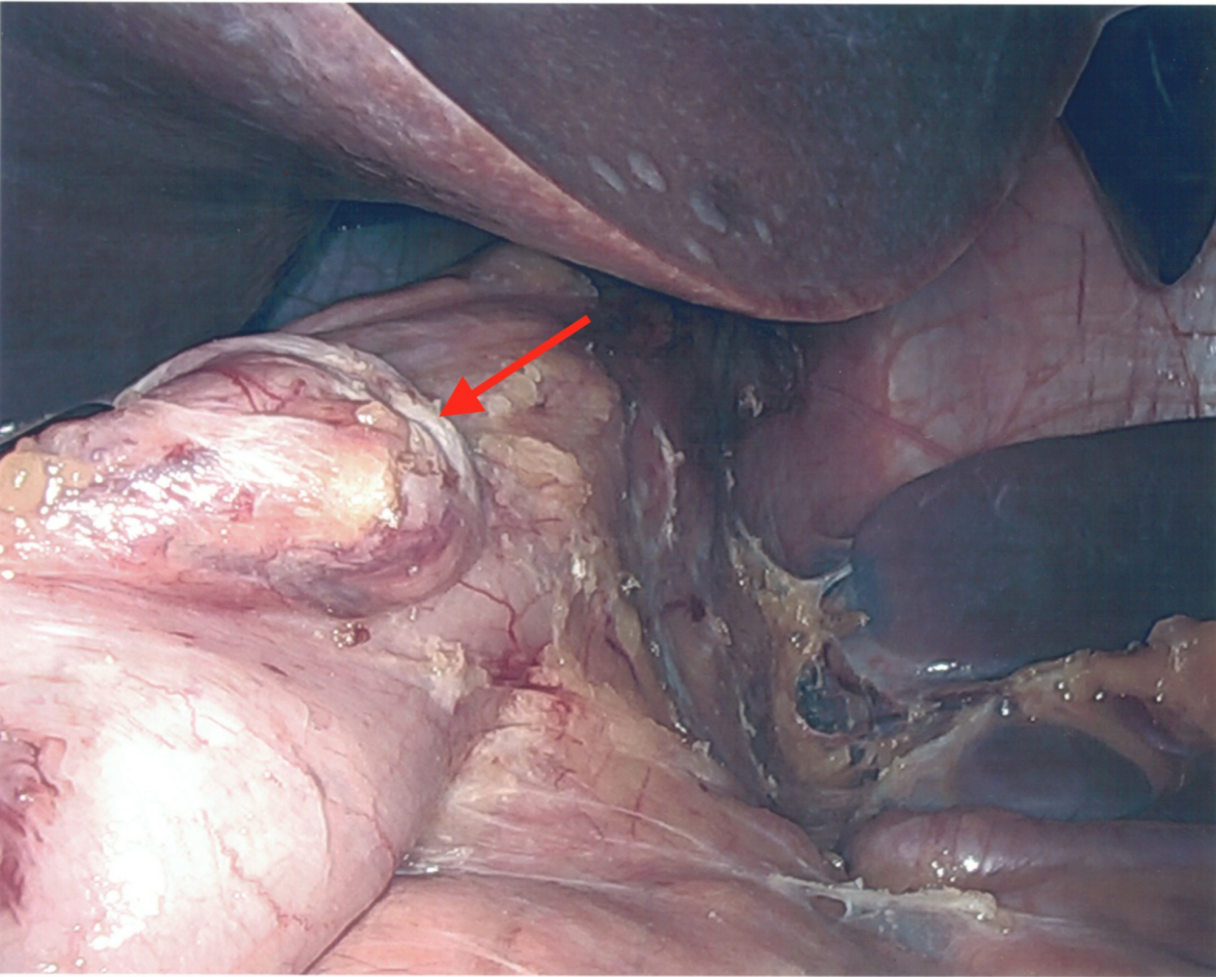
Gastric diverticulum located on the greater curvature of the stomach.

The vessels along the greater curvature of the stomach, including the short gastric vessels, were ligated creating a window of visualization of the posterior wall of the fundus. The 2.4-cm gastric diverticulum was identified 3 cm distal to the angle of His, oriented with the base towards the greater curvature of the stomach, favorable to resection concurrent with a sleeve gastrectomy as seen in Figure 5.

**Figure 6:**
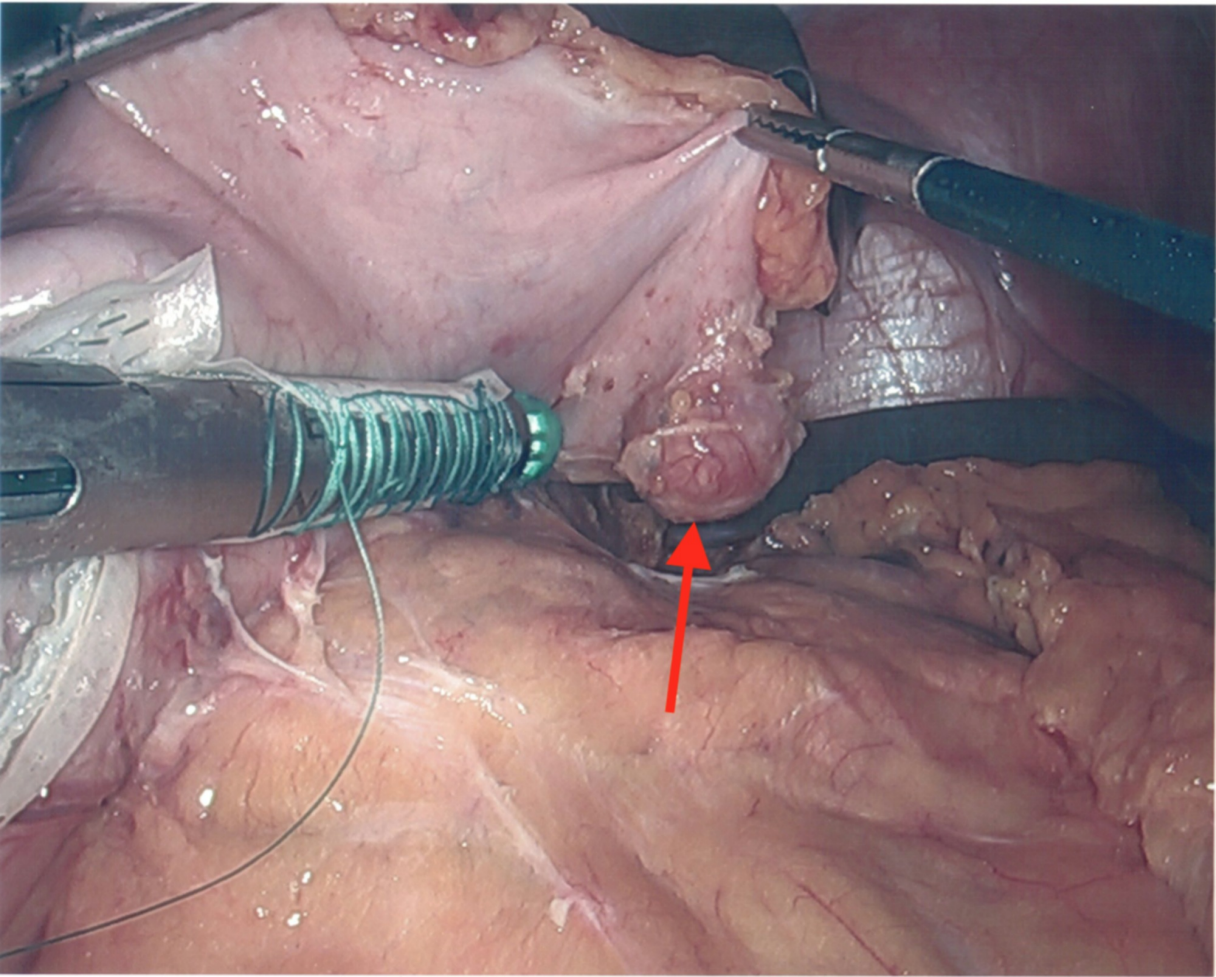
The gastric diverticulum after vessels were divided and sealed along the greater curvature of the stomach.

**Figure 7:**
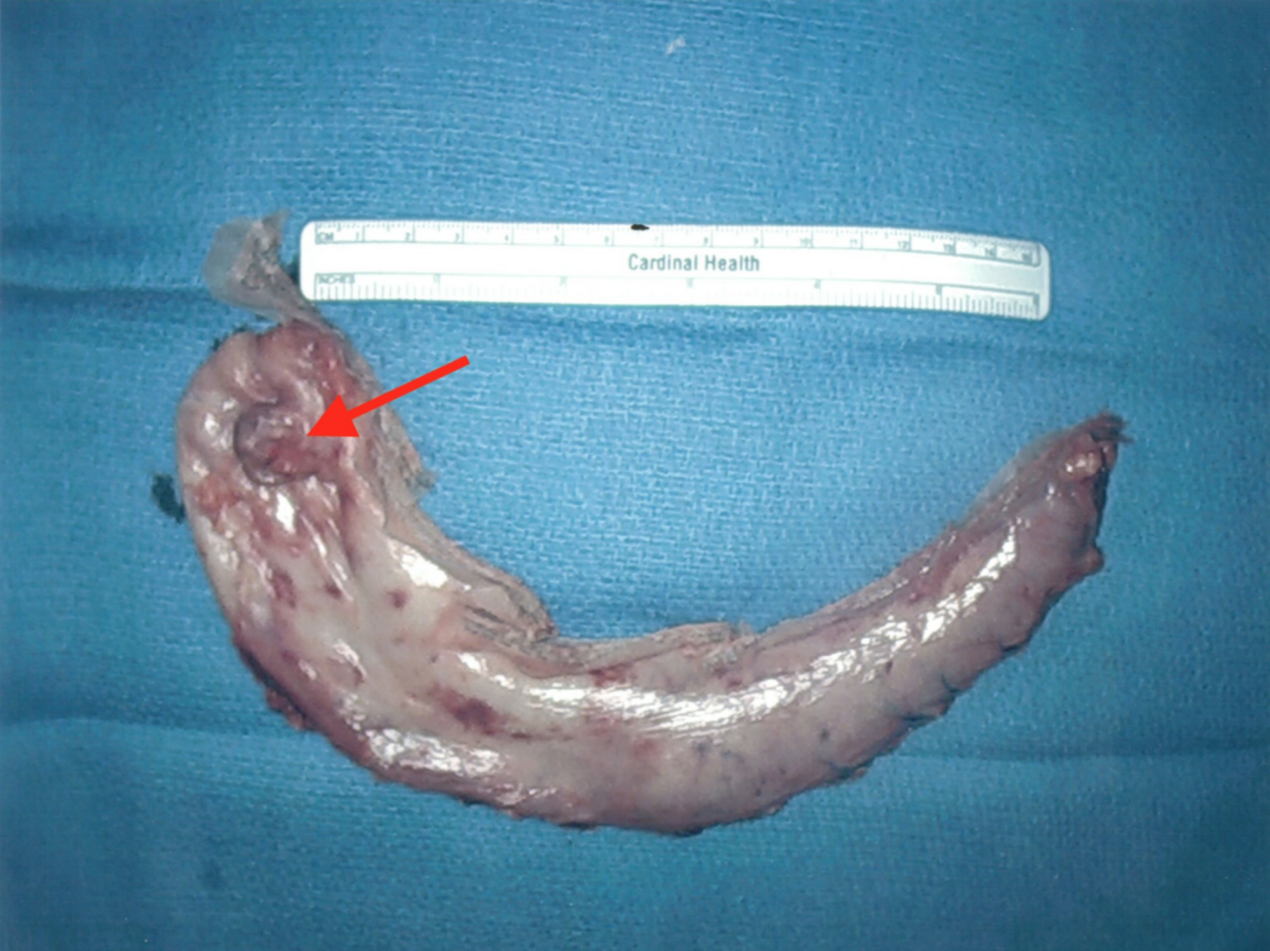
Resected greater curvature and fundus of the stomach including the gastric diverticulum.

**Figure 8:**
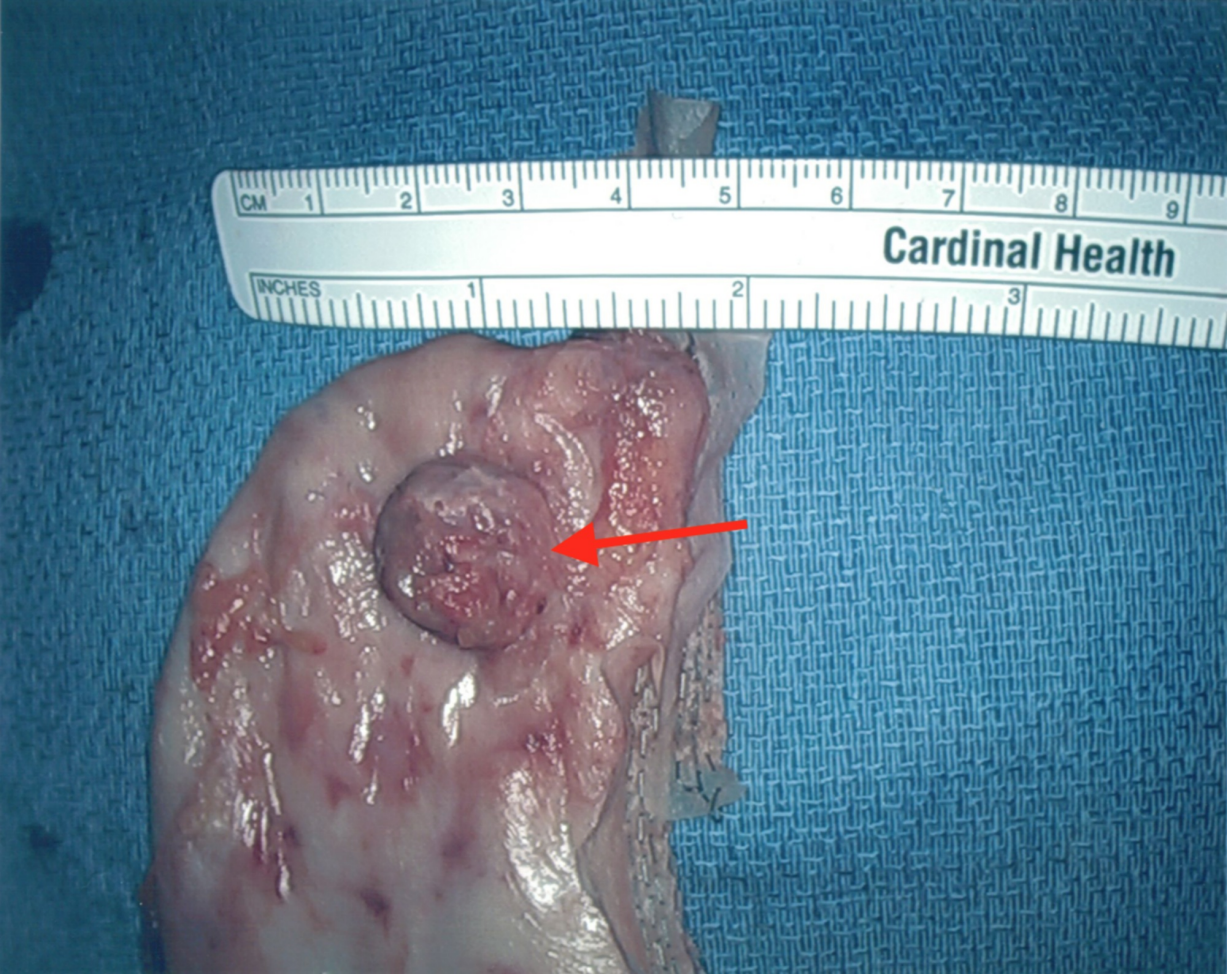
An anterior view of the gastric diverticulum and resected stomach.

**Figure 9:**
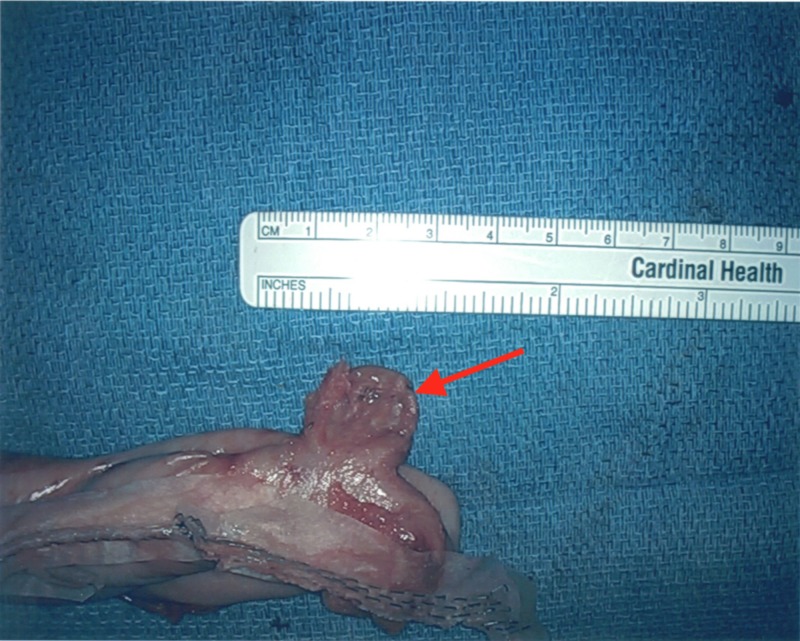
A lateral view of the gastric diverticulum and resected stomach.

The stomach was marked 6 cm proximally to the pylorus. The vessels along the greater curvature of the stomach were dissected and sealed using a Harmonic scalpel. The stomach was lifted and all posterior attachments to the pancreas were sharply divided as seen in Figure 6. A 42-French bougie was placed. The stomach was stapled and divided using Ethicon Flex triple staple line power stapler with one black and four green 60 mm Gore bioseamguard reinforced staple loads to resect the greater curvature and fundus of the stomach including the gastric diverticulum and a 3-cm margin of unaffected tissue as seen in Figures 7-9. The 15 mm trocar and specimen were removed. The bougie was removed and an intraoperative endoscopy was performed using a 5 mm Olympus Ultrathin gastroscope, revealing no areas of stenosis, leak, bleeding, or staple malfunction.

The trocars were removed, and the 15-mm incision was closed laparoscopically. Local anesthetic was injecting at the port sites. The skin was closed with staples, and sterile dressings were applied. The patient tolerated the procedure well, and there were no postoperative complications.

**Figure 10:**
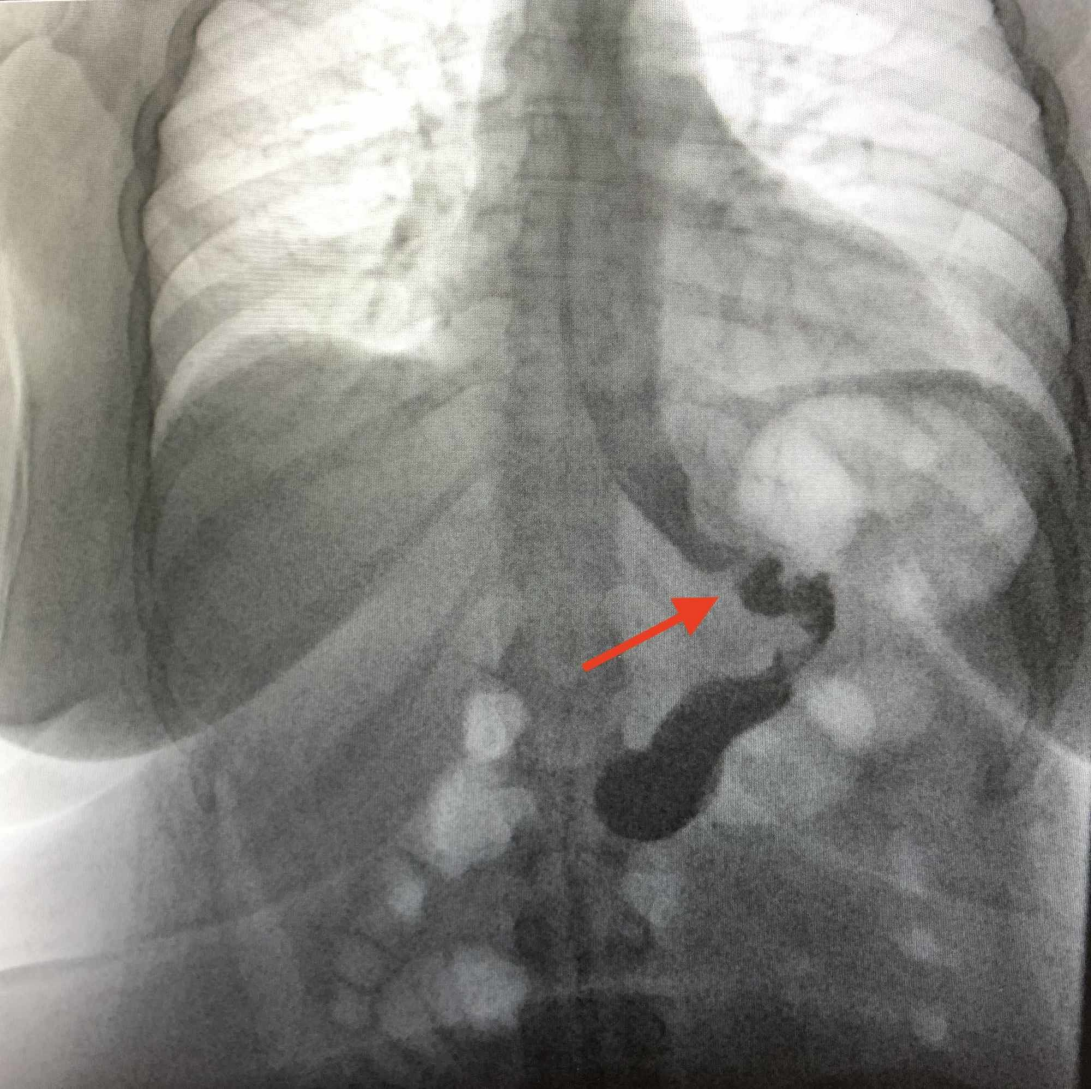
Postoperative UGI without evidence of stenosis or leak of gastric content. UGI: Upper gastrointestinal series

The patient’s postoperative course included pain management and DVT prophylaxis including heparin administration, SCD boots, and ambulation every four hours. On postoperative day 1, the patient underwent a UGI, which revealed normal gastric emptying without evidence of obstruction or extravasation as evidenced in Figure 10.

The patient was discharged on postoperative day 1 with no complications. One week after the discharge, the patient followed up in office with no complaints. She was tolerating liquids, taking her antacids as prescribed, and attending appointments with a registered dietician. Two weeks later, the patient returned to the office with no postoperative complaints or complications. At the three-month post-surgical appointment, the patient was tolerating regular diet and no longer requiring proton pump inhibitors. She lost 45 pounds since her consultation, currently weighing 215 pounds with a BMI of 38. The patient is scheduled for a six-month postoperative appointment at which she will obtain routine postoperative labs including a CBC, liver function test, kidney function test, vitamin B6, vitamin B12, folate, albumin, and prealbumin levels. At this appointment, she will also be counseled on discontinuing her antacids and encouraged to continue taking vitamin supplementation daily. 

## Discussion

Due to the rare incidence of gastric diverticula, few studies have been reported on LSGs with simultaneous management of a gastric diverticulum. Our case supports the prior report outlining the resection of a gastric diverticulum concurrently with a sleeve gastrectomy in two case studies [6]. Sleeve gastrectomies with concurrent diverticulectomies have also been reported as a procedure to alleviate chronic nausea after laparoscopic gastric imbrication [7]. Goitein et al. describe a symptomatic gastric diverticulum after a Roux-en-Y gastric bypass, located along the gastric remnant staple line, which was successfully resected laparoscopically [8].

Our patient presented with chronic GERD, which was resistant to proton pump inhibitor treatment. The goal of the surgery was to resect the diverticulum while performing a bariatric surgical procedure. As discussed above, the patient underwent several studies before her surgery to localize the diverticulum. However, these studies were contradictory. The UGI was read as a 2.4-cm diverticulum on the lesser curvature of the stomach. The CT scan was read as normal, with no mention of a gastric diverticulum. The EGD reported a small diverticulum in the gastric cardia region.

Due to the contradictory reports of size and location of the diverticulum on preoperative studies, the patient was consented for several possible procedures depending on the true location of the diverticulum. The primary goal of this procedure was to resect the diverticulum with appropriate margins. The secondary goal was to perform a concurrent bariatric procedure. If the diverticulum was not located in a position conducive to a sleeve gastrectomy, the patient was prepared to undergo diverticulectomy alone or in combination with a Roux-en-Y gastric bypass. Due to the location of our patient’s diverticulum along the greater curve of the stomach, we were able to fully resect the diverticulum while performing a sleeve gastrectomy.

## Conclusions

Morbidly obese patients undergoing a laparoscopic gastric diverticulectomy with concurrent LSG should undergo thorough preoperative workup and preparation including studies (CT scan, EGD, and UGI) to help establish the location of the diverticulum. During the operation, the surgeon must locate the diverticulum and use sound intraoperative judgment to determine whether diverticulectomy with a sleeve gastrectomy is feasible. Laparoscopic gastric diverticulectomy with concurrent LSG is a safe and effective procedure as part of a multidisciplinary treatment approach to morbid obesity with an anatomically favorable symptomatic gastric diverticulum. This strong approach treats a patient’s gastric symptoms as well as his or her health and wellness beyond the gastrointestinal system. 
